# Relapses in canine leishmaniosis: risk factors identified through mixed-effects logistic regression

**DOI:** 10.1186/s13071-024-06423-1

**Published:** 2024-08-22

**Authors:** Juliana Sarquis, Letícia Martins Raposo, Carolina R. Sanz, Ana Montoya, Juan Pedro Barrera, Rocío Checa, Blanca Perez-Montero, María Luisa Fermín Rodríguez, Guadalupe Miró

**Affiliations:** 1 Animal Health Department, Faculty of Veterinary Medicine, Complutense University of Madrid, Madrid, Spain; 2 Department of Quantitative Methods, Universidade Federal Do Estado Do Rio de Janeiro, Rio de Janeiro, Brazil; 3 Departament of Medicine and Animal Surgery, Faculty of Veterinary Medicine, Complutense University of Madrid, Madrid, Spain

**Keywords:** Leishmania, Leishmaniosis, Relapses, Treatment, Protein electrophoresis, Dysproteinemia, Logistic regression, Retrospective study

## Abstract

**Background:**

Canine leishmaniosis (CanL), caused by *Leishmania infantum*, is an important vector-borne parasitic disease in dogs with implications for human health. Despite advancements, managing CanL remains challenging due to its complexity, especially in chronic, relapsing cases. Mathematical modeling has emerged as a powerful tool in various medical fields, but its application in understanding CanL relapses remains unexplored.

**Methods:**

This retrospective study aimed to investigate risk factors associated with disease relapse in a cohort of dogs naturally infected with *L. infantum*. Data from 291 repeated measures of 54 dogs meeting the inclusion criteria were included. Two logistic mixed-effects models were created to identify clinicopathological variables associated with an increased risk of clinical relapses requiring a leishmanicidal treatment in CanL. A backward elimination approach was employed, starting with a full model comprising all potential predictors. Variables were iteratively eliminated on the basis of their impact on the model, considering both statistical significance and model complexity. All analyses were conducted using R software, primarily employing the lme4 package, and applying a significance level of 5% (*P* < 0.05).

**Results:**

This study identified clinicopathological variables associated with an increased risk of relapses requiring a leishmanicidal treatment. Model 1 revealed that for each 0.1 increase in the albumin/globulin ratio (A/G) ratio, the odds of requiring treatment decreased by 45%. Conversely, for each unit increase in the total clinical score (CS), the odds of requiring treatment increase by 22–30%. Indirect immunofluorescence antibody test (IFAT) was not a significant risk factor in model 1. Model 2, incorporating individual albumin and globulins values, showed that dogs with high IFAT titers, hyper beta-globulinemia, hypoalbuminemia, anemia, and high CS were at increased risk of relapse. Both models demonstrated a good fit and explained a substantial amount of variability in treatment decisions.

**Conclusions:**

Dogs exhibiting higher CS, dysproteinemia, anemia, and high IFAT titers are at increased risk of requiring leishmanicidal treatment upon clinical relapse in CanL. Regular monitoring and assessment of risk factors prove essential for early detection of relapses and effective intervention in CanL cases. The contrasting findings between the two models highlight the complexity of aspects influencing treatment decisions in this disease and the importance of tailored management strategies to improve outcomes for affected dogs.

**Graphical Abstract:**

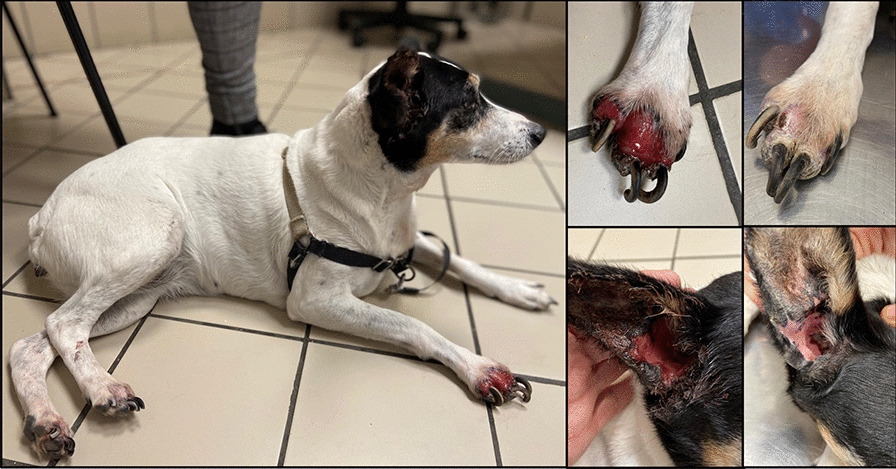

**Supplementary Information:**

The online version contains supplementary material available at 10.1186/s13071-024-06423-1.

## Background

Canine leishmaniosis (CanL), caused by *Leishmania infantum*, is among the most important vector-borne parasitic diseases of dogs, considered the main peridomestic reservoir of human infection for phlebotomine sand flies [1]. Despite advances in diagnosis and treatment, the management of CanL remains challenging due to the complexity of the disease, which often involves chronic, relapsing cases characterized by intermittent clinical manifestations and varying responses to therapy [2, 3]. Relapses, defined as the recurrence of clinical signs and/or clinicopathological abnormalities after an initial period of clinical improvement [4–6], represent a major concern in the clinical management of CanL. These relapses not only contribute to prolonged morbidity in sick dogs, but also represent challenges in terms of treatment efficacy and disease control [3]. Early identification of relapses is therefore crucial for optimizing therapeutic strategies and improving long-term outcomes in sick dogs.

In recent years, mathematical modelling approaches have emerged as powerful tools for predicting disease outcomes and guiding clinical decision-making in various medical fields, including infectious diseases [7]. By integrating epidemiological data, clinicopathological parameters, and host–parasite features, mathematical models offer the potential to identify predictive factors associated with infection and disease and to develop prognostic tools and individualized treatment strategies.

In the field of leishmaniosis, mathematical modeling has been used to identify risk factors for infection or seropositivity [8, 9], to explore genetic susceptibility to infection [10], to identify biomarkers for therapy outcomes [11], and to evaluate methods of control for human leishmaniasis [7], among others. However, to the best of our knowledge, no published studies have delved into the risk factors associated with disease relapse in CanL using mathematical models.

In this study, we aimed to identify potential risk factors of relapse among clinicopathological parameters commonly used in the monitoring of dogs with CanL, employing mixed-effects logistic regression modeling.

## Methods

### Inclusion criteria and selection of records

**Fig. 1:**
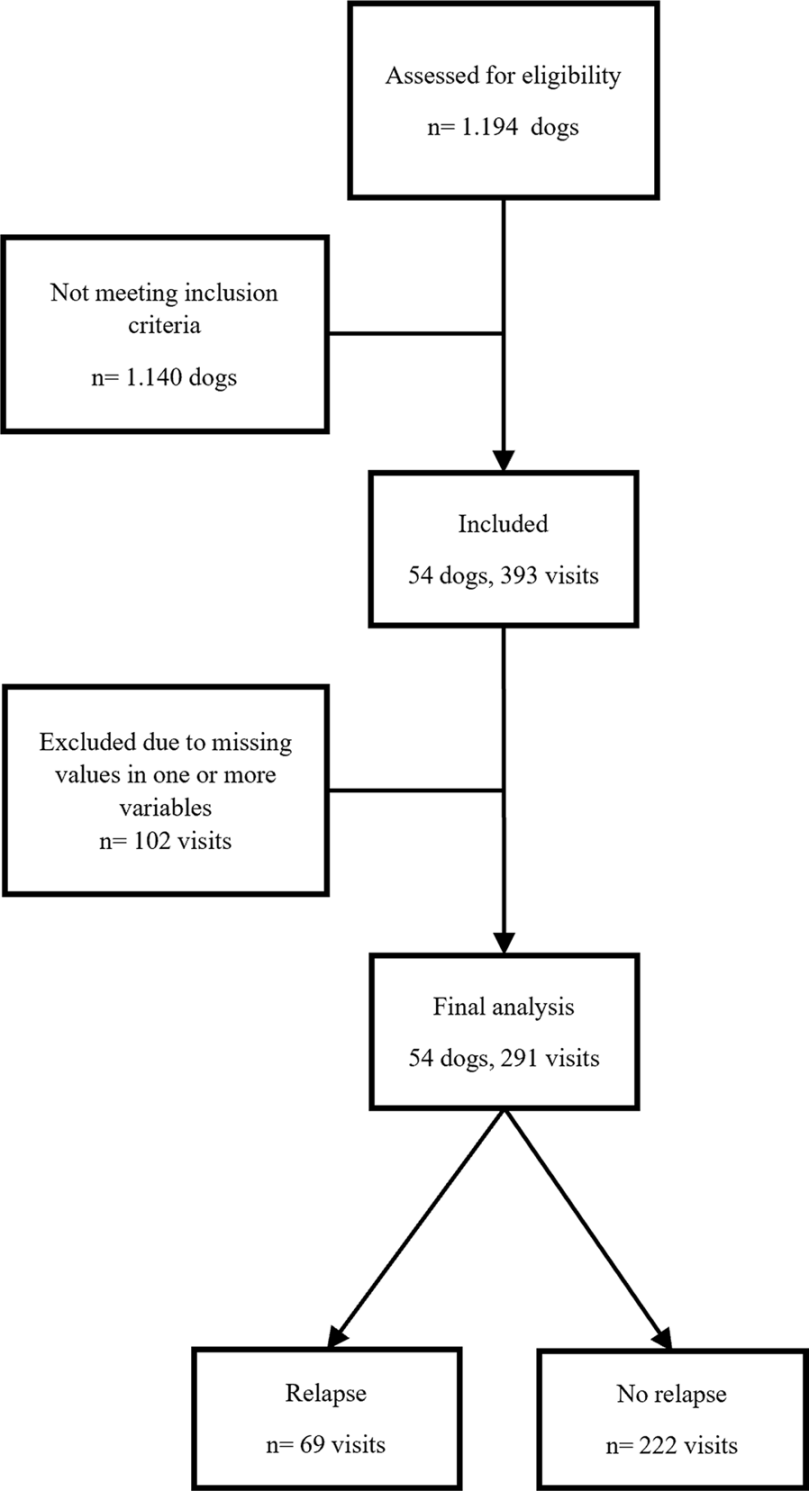
Flow diagram of the inclusion procedure of the study

For this study, we assessed an electronic database of 1194 cases of canine leishmaniosis that sought consultation at the Veterinary Teaching Hospital of the Universidad Complutense de Madrid between 2010 and 2022 to identify those with the following inclusion criteria: (1) a confirmed diagnosis of *L. infantum* infection by indirect immunofluorescence antibody test (IFAT) or polymerase chain reaction (PCR) (IFAT ≥ 1:200 and/or PCR positive), (2) data on clinical signs and physical examination, (3) hematology and (4) plasma protein electrophoresis (PE) results, and (5) information about treatment regimen. Dogs affected by other vector-borne diseases (e.g., ehrlichiosis, anaplasmosis, babesiosis, dirofilariosis) were excluded to mitigate potential confounding factors, as the clinical signs and clinicopathological abnormalities associated with these conditions closely mimic those of leishmaniosis. Of those, 54 cases met the inclusion criteria and were selected for the study (Fig. 1).

### Data extraction and processing

The medical records of the 54 dogs were analyzed to extract clinicopathological data obtained during each visit to our infectious diseases clinical unit. Raw data were stored in a table in.xlsx format. Demographic data, clinical signs, and physical examination findings were carefully recorded using a scoring system (Additional file 1: Table S1) ranging from 0 to 3 (indicating low to high severity) to obtain a total clinical score (CS) for each visit (maximum score of 74) (adapted from [12]).

Data on laboratory parameters were also extracted, including indirect immunofluorescence antibody test (IFAT) titers, hematocrit (%) (HCT), white blood cell count (× 10^3^/$\mu$ l) (WBC) and platelet count (× 10^3^/$\mu$ l) (PLT), total plasma proteins (g/dl), albumin (g/dl) (ALB), alpha-1 (g/dl), alpha-2 (g/dl), beta (g/dl), and gamma (g/dl) globulins, and albumin/globulin (A/G) ratio. Finally, information regarding the treatment regimen at each visit was also recorded. A relapse was defined as the initiation of a new cycle of treatment with meglumine antimoniate or miltefosine due to worsening of clinical signs and/or laboratory parameters [5, 6]. Dogs that did not receive a new treatment were considered clinically stable (no relapse).

### Selection of variables

The outcome variable was binary, representing the receipt of treatment (no/yes) upon disease relapse. The clinicopathological parameters selected as risk factors associated with the likelihood of receiving treatment were selected on the basis of previous research [4, 12–15]. Numerical risk factors considered in the analysis included age, total proteins, ALB, alpha-1, alpha-2, beta, and gamma-globulins, A/G ratio, HCT, WBC, PLT, and CS. Categorical variables consisted of sex (male or female), breed (mongrel or purebred), and IFAT (negative: < 1:200, low positive: 1:200–1:400, medium positive: 1:800–1:1600, or high positive: ≥ 1:3200). IFAT cut-offs were defined according to values established by the laboratory [4, 16–18].

### Statistical analysis and logistic regression model

For descriptive statistics, the median and interquartile ranges were calculated for all numeric data, given the non-normal distribution observed in the dataset. Additionally, frequency distributions were analyzed for categorical data.

#### Logistic mixed-effects model

The study employed a generalized linear mixed model (GLMM) framework to assess the impact of various predictors on the response variable treatment, assuming a binomial distribution. The GLMM accommodates repeated measurements on the same subject by incorporating a subject-specific random effect into the model, thereby capturing unobserved subject-specific characteristics. The logistic mixed-effects regression model is utilized to model binary outcome variables, wherein the log odds of the outcomes are expressed as a linear combination of the risk factors when both fixed and random effects are accounted for. The rationale for using repeated measures was the chronic feature of CanL and the fact that relapses can occur at any given time due to multiple reasons [19, 20].

Since regression models can be influenced by correlated variables, we tested for multicollinearity using the variance inflation factor (VIF) to ensure that explanatory variables were not highly correlated. Such a scenario would impede the accurate interpretation of the odds ratio (OR), as the outcome would no longer be solely influenced by an individual variable. All variables incorporated into the models demonstrated low VIF values (< 2) [21]. The dataset consisted of 291 observations nested within 54 subjects. Two models were developed:

**Model 1** encompassed a full generalized mixed-effects model on treatment, aiming to incorporate one random effect (the intercepts of subjects) and nine fixed effects, namely, sex, breed, age, IFAT, A/G, HCT, WBC, PLT, and CS.

**Model 2** encompassed a full generalized mixed-effects model based on treatment, attempting to integrate one random effect (the intercepts of subjects), and 13 fixed effects, namely, sex, breed, age, IFAT, ALB, alpha-1, alpha-2, beta, and gamma globulins, HCT, WBC, PLT, and CS.

Model fitting utilized maximum likelihood estimation with adaptive Gauss–Hermite quadrature. The aim of model selection was to identify significant predictors of the dependent variable while preventing overfitting. A backward elimination approach was employed, starting with a full model comprising all potential predictors. Variables were iteratively eliminated on the basis of their impact on the model, considering both statistical significance and model complexity. At each step, the variable with the highest *P*-value exceeding 0.15 and the least improvement in Akaike information criterion (AIC) was removed. AIC balances model complexity and goodness-of-fit, ensuring a trade-off between simplicity and predictive accuracy. The remaining variables were used to refine the model, and this process was repeated until all remaining variables had *P*-values below 0.15 and made meaningful contributions to the model according to the AIC [22]. After model fitting, an analysis of variance (ANOVA) was performed to compare the explained variance of each model generated during the backward elimination process. This provided a quantitative evaluation of how the model improved with each iteration and supported the selection of the final model.

The variance explained by the models was quantified as *R*^2^ marginal (indicating the variance explained by fixed effects) and *R*^2^ conditional (indicating the variance explained by both random and fixed effects), following a method tailored for generalized linear mixed-effects models [23]. All analyses were conducted using R software [24], employing the lme4 package [25], and applying a significance level of 5% (*P* < 0.05).

## Results

Of the 54 dogs included in the study, 32 (59.2%) were male and 22 (40.7%) female, 18 (33.3%) were mongrel dogs and 37 (66.6%) were purebred, and their ages ranged from 1 to 13 years (median 6 years; IQR 4.30, 8.0). The number of visits for each dog ranged from 1 to 14 visits (median 4 visits; IQT 3, 7), resulting in a total of 291 visits. Follow-up recorded for the same dog ranged from 4.2 months to 9.4 years (median 1.96 years; IQR 0.9, 3.6 years).

Total clinical scores ranged between 0 and 18 (median 1; IQR 0, 3). While clinical signs were present at 151 visits (51.9%), dogs received leishmanicidal treatment at only 69 visits (23.7%). Medium IFAT titers (1:800–1:1600) were detected at most visits (128/291, 43.9%), followed by low positive titers (1:200–1:400) (99/291, 34%) and negative titers (< 1:200) (51/291, 18.5%). High antibody titers (≥ 1:3200) were detected in 13/291 visits (4.4%). The most common laboratory abnormality detected was hyper-gammaglobulinemia (49.8%), followed by decreased A/G ratio (38.1%), hyperproteinemia (33.3%), and leukopenia (33.3%) (Table 1).

**Table 1:** Distribution of laboratory parameters of the dogs included in the study

*n* = 291	RI	Median (IQR)	Low	Normal	High
HCT (%)	37–55	43 (38, 48)	44 (15.1%)	233 (80%)	14 (4.8%)
WBC (× 10^3^/µl)	6–17	7.08 (5.49, 9.38)	97 (33.3%)	187 (64.2%)	7 (2.4%)
PLT (× 10^3^/µl)	200–500	249 (183, 300)	86 (29.5%)	195 (67%)	10 (3.4%)
Total proteins (g/dl)	5.8–7.5	7.2 (6.6, 8.0)	14 (4.8%)	180 (61.8%)	97 (33.3%)
Albumin (g/dl)	2.7–4.6	3.17 (2.75, 3.5)	66 (22.7%)	225 (77.3%)	0 (0%)
Alpha-1 globulins (g/dl)	0.2–0.5	0.30 (0.25, 3.50)	7 (2.4%)	273 (93.8%)	11 (3.8%)
Alpha-2 globulins (g/dl)	0.3–1.1	0.54 (0.42, 0.68)	19 (6.5%)	263 (90.3%)	9 (3.1%)
Beta globulins (g/dl)	1.3–2.7	1.60 (1.32, 1.91)	68 (23.3%)	205 (70.4%)	18 (6.2%)
Gamma globulins (g/dl)	0.5–1.2	1.22 (0.88, 2.16)	8 (2.7%)	136 (47.4%)	145 (49.8%)
A/G ratio	0.7–1.9	0.86 (0.54, 1.07)	111 (38.1%)	180 (61.8%)	0 (0%)

*RI* reference intervals for dogs according to the laboratory (UCM), *IQR* interquartile range, *HCT* hematocrit, *WBC* white blood cells, *PLT* platelets

### Models

In this study, we aimed to investigate potential risk factors associated with clinical relapses requiring leishmanicidal treatment in CanL among a set of nine (model 1) and 13 (model 2) clinicopathological variables. A backward elimination approach was employed, starting with a full model comprising all potential predictors. Variables were iteratively eliminated on the basis of their impact on the model, considering both statistical significance and model complexity. At each step, the variable with the highest *P*-value exceeding 0.15 and the least improvement of AIC was removed. Given that the A/G ratio and individual globulins values are highly correlated variables, we created two separate models to avoid redundancy.

Model 1 retained only the A/G ratio and CS as fixed effects. In this model, the A/G ratio was inversely associated with the outcome. Notably, each increase of 0.1 in the A/G ratio was associated with a 45% decrease in the odds of receiving treatment due to a clinical relapse (OR 0.55, 95% CI 0.45–0.65, *P* < 0.001) (Table 2). Furthermore, the CS of the subjects was significantly associated with treatment (OR 1.22, 95% CI 1.08–1.37,* P* = 0.001), with a 22% increase in the odds of receiving treatment per each unit increase in the CS. Table 2 presents the odds ratios (OR) along with their corresponding 95% confidence intervals (CI) and *P*-values for both predictor variables.

**Table 2:** Odds ratio of the generalized linear mixed-effects found for model 1 on treatment with CS and A/G ratio included in the model

*Predictors*	Treatment		
	*Odds ratios*	*CI*	*P*
(Intercept)	10.87	3.03–38.98	< 0.001*
A/G	0.55^a^	0.45–0.65	< 0.001*
CS	1.22	1.08–1.37	0.001*
Random effects			
*σ*^2^	3.29		
*τ*_00_ _subject_	0.21		
*N* _subject_	54		
Observations	291		
Marginal *R*^2^/Conditional *R*^2^	0.621 / 0.644		
AIC	188.665		

^a^For every 0.10 increase in A/G

*CS* total clinical score, *CI* confidence interval, *P <0.05

Model fit was assessed using statistical measures, including marginal *R*^2^, conditional *R*^2^, and Akaike information criterion (AIC). Marginal *R*^2^ represents the variance explained by the fixed effects in the model and indicates how well the model fits the observed data. It is calculated as the proportion of variance explained by the fixed effects alone, without considering the random effects. In our study, model 1 explained 62% of the variance in the data (marginal *R*^2^), indicating a substantial amount of variability in the outcome variable (treatment receipt upon disease relapse) accounted by the predictor variables included in the model. Conditional *R*^2^ represents the variance explained by both the fixed and random effects in the model and provides a more comprehensive measure of model fit. It is calculated as the proportion of variance explained by both the fixed and random effects combined. In our study, model 1 explained 64% of the total variance when accounting for both fixed and random effects (conditional *R*^2^) (Table 2), indicating a substantial overall fit of the model to the data.

Model 2 retained IFAT, ALB, beta globulins, HCT, and CS as fixed effects. The second model revealed that dogs with high IFAT titers exhibit nearly 18 times greater odds (OR 17.98, 95% CI 1.64–197.28, *P* = 0.018) of requiring treatment compared with dogs with a negative IFAT (Table 3). Additionally, higher levels of beta globulins were associated with an increased chance of relapse (OR 1.10, 95% CI 1.03–1.17, *P* = 0.002), with every 0.1 increase in beta globulins increasing the odds of receiving treatment by 10%. Notably, the CS exhibited a significant positive association with the chance of treatment, with each unit increase in the CS associated with a 30% increase in the odds of receiving treatment (OR 1.30, 95% CI 1.15–1.48, *P* < 0.001). In contrast, higher levels of ALB (OR 0.89, 95% CI 0.82–0.96, *P* = 0.004) and HCT were associated with decreased odds of receiving treatment. (OR 0.93, 95% CI 0.88–0.99, *P* = 0.014). Specifically, for every 0.1 increase in albumin values, the odds of receiving treatment reduced by 11%, while for each unit (1.0) increase in HCT values they decreased by 7%. Lastly, model 2 explained 64% of the variance in the data (marginal *R*^2^) and 66% of the total variance when accounting for both fixed and random effects (conditional *R*^2^) (Table 3).

**Table 3:** Odds ratio of the generalized linear mixed effects found for model 2 on treatment, with globulins fractions included in the model, but not the A/G ratio

*Predictors*	Treatment		
	*Odds ratios*	*CI*	*P*
(Intercept)	5.75	0.12–270.92	0.374
IFAT (low positive)	0.98	0.16–6.17	0.982
IFAT (medium positive)	4.62	0.90–23.71	0.066
IFAT (high positive)	17.98	1.64–197.28	0.018*
ALB^a^	0.89	0.82–0.96	0.004*
Beta globulins^a^	1.10	1.03–1.17	0.002*
HCT	0.93	0.88–0.99	0.014*
CS	1.30	1.15–1.48	< 0.001*
Random effects			
*σ*^2^	3.29		
*τ*_00_ _subject_	0.16		
*N*_subject_	54		
Observations	291		
Marginal *R*^2^/conditional *R*^2^	0.643/0.659		
AIC	193.377		

^a^For every 0.10 increase in ALB or beta globulins.

*IFAT* indirect immunofluorescence antibody test, *ALB* albumin, *HCT* hematocrit (%), *CS* total clinical score, *CI* confidence interval,  *P < 0.05

## Discussion

The results of the present study revealed important clinicopathological variables associated with an increased risk of clinical relapses requiring leishmanicidal treatment in dogs with CanL. The study cohort comprised 54 dogs, predominantly male purebreds with a median age of 6 years. The association of breed, sex, and age with infection or disease has been investigated in several studies, with conflicting results [26–31]. In our study, these variables were eliminated from both models in the backwards process, as they did not significantly improve the model’s predictive capacity in the presence of other more important variables.

Clinical signs were observed in more than half of the visits (51.9%), but a relapse was only present in one-third of them (23.7%). This is expected, as some clinical signs in CanL, such as uveitis and vasculitis, have chronic presentation and can take several weeks or even months to resolve. Nevertheless, the CS, derived from the sum of the presence and severity of various clinical signs, remained an integral part of both models, indicating that dogs with higher CS were more likely to require leishmanicidal treatment. Specifically, for every unit increase in the CS, the odds of requiring treatment increased from 22% to 30% (model 1 and model 2, respectively). This result underscores the potential value of employing a clinical scoring chart in the ongoing monitoring of dogs with CanL. While previous researchers have proposed different clinical scoring systems [12, 14, 32, 33], the absence of a validated scoring system for use in both clinical practice and research persists [18]. As pointed out by Meléndez-Lazo et al. (2018), an ideal scoring model should encompass relevant clinical signs and laboratory parameters, incorporating varying weights among parameters that could influence prognosis and survival, particularly those linked to renal function. Employing mathematical models could aid in the development of such scoring systems in future studies.

Regarding clinicopathological findings, the most common alterations detected were hyper-gamma globulinemia (49.8%), decreased A/G ratio (38.1%), hyperproteinemia (33.3%), and leukopenia (33.3%), in agreement with previous research [15, 18, 34, 35]. In contrast, alpha‐1 and alpha-2 globulins were elevated in only a small number of visits (3.8% and 3.1%, respectively) (Table 1). One study found a significant increase in alpha‐2 globulins in dogs with leishmaniosis, however, dogs were not treated and were followed for 1 month only [13]. Paltrinieri et al. (2016) also described a moderate increase in alpha-2 globulins in early stages of the disease. The stage of the disease at which the dogs were assessed in each study and/or differences in PE techniques used could explain this discrepancy.

In the first model, the A/G ratio emerged as a significant predictor of relapse. Notably, each increase of 0.1 in the A/G ratio was associated with a 45% decrease in the odds of receiving treatment due to a clinical relapse. Protein electrophoresis has been proven to be extremely useful for the diagnosis and monitoring of CanL and is routinely performed in clinical practice [15, 32, 36, 37]. In fact, previous studies demonstrated that this technique may show abnormalities very early during the course of the disease and even before the onset of overt clinical signs [13, 32, 35, 36, 38, 39].

Interestingly, IFAT titers were not statistically significant, and therefore were not retained in model 1. This indicates that in the presence of a low A/G ratio and clinical signs suggestive of CanL, the IFAT titer may not be a robust predictor of relapse. Even though previous research has established an association between high antibody titers and clinical disease [2, 15, 40], practitioners must be aware of IFAT’s limitations when using it for monitoring treatment efficacy or detecting clinical relapses. For instance, serology may not be a useful parameter in the short term as antibody titers may take up to 6 months to substantially decrease [12, 15, 38, 41]. Additionally, the dynamics of IFAT titers vary on the basis of the dog’s inherent response to treatment. Dogs with a favorable response typically exhibit a reduction in antibody titers [38, 42, 43], whereas those with suboptimal responses may maintain chronically elevated titers or experience only minor or temporary decreases [4, 44–46]. Furthermore, an increase in antibody levels may occur in the presence of other infections or non-infectious diseases such as endocrinopathies or neoplasia that trigger the multiplication of *Leishmania* parasites [47]. Consequently, practitioners should not rely solely on an elevation in IFAT titers to initiate leishmanicidal treatment, as evidenced by the performance of our models incorporating other variables.

The substitution of the A/G ratio with globulins fractions in model 2 led to interesting results. In the absence of this variable, the presence of high antibody titers (> 1:1600) was significantly associated with relapse. Low albumin, considered a negative prognostic factor in CanL [48], was a common finding (22.7%) in our study, and an increase in its values was associated with a decreased risk of relapse. In contrast, hyperbetaglobulinemia was associated with an increased risk, despite its relatively low frequency in the study (6.2%). Surprisingly, gamma-globulins were not retained in model 2, despite hypergammaglobulinemia being the most common laboratory disorder detected (49.8%). One explanation for this finding is the moderate correlation between gamma-globulins and HCT (*r* = −0.53) (data not shown), the last being retained in the model. When two variables are highly correlated, the model may have difficulty distinguishing the unique contributions of each variable to the outcome and may choose to retain only one of them to avoid multicollinearity issues. Therefore, considering the possibility that most anemic dogs also had hypergammaglobulinemia, the last was removed in the stepwise process for not significantly contributing to the model’s predictive capability in the presence of the other variables.

In model 2, HCT also emerged as a significant predictor of relapse. Anemia, present in 15.1% of visits in this study, is one of the most frequent clinicopathological abnormalities detected in CanL and is often associated with the presence of clinical signs [13, 49]. However, in the first model, HCT did not significantly enhance the model’s predictive capacity, indicating that in the presence of a low A/G ratio and clinical signs, the presence of anemia is a less critical factor to consider before initiating leishmanicidal treatment in suspected relapse cases.

The decision to develop two separate models, one including the A/G ratio and the other incorporating individual albumin and globulins values, was driven by the high collinearity between these variables. Furthermore, considering that determining all protein fractions may not always be feasible due to equipment availability or cost constraints, opting for two models seemed plausible. The contrasting findings between the two models highlight the need for a comprehensive approach to risk assessment in CanL clinical management.

The assessment of model fit using statistical measures such as the marginal *R*^2^, conditional *R*^2^, and AIC provided insights into the performance of the models. The relatively high marginal *R*^2^ and conditional *R*^2^ values indicate that the models explained a substantial amount of variability in the outcome variable, accounting for both fixed and random effects.

It is important to recognize the limitations of our study, including the relatively small sample size and the retrospective nature of the data analysis. Future studies with larger sample sizes and prospective designs are warranted to validate our findings and enhance our understanding of the factors influencing relapses in CanL. Additionally, the characteristics of the study population being limited to dogs referred to a specialist in infectious and parasitic diseases may affect the model’s performance in other populations of dogs [50]. Lastly, the presence of other diseases can potentially influence the performance of our model, and this should be taken into consideration in future studies. Therefore, in suspected relapses, the presence of other infectious, metabolic, or neoplastic diseases that could impair the immune system should always be considered before starting a leishmanicidal treatment, even in the presence of the clinicopathological alterations considered significant in this study. Focusing on CanL as an isolated disease may lead to poor clinical management and treatment response.

Early detection of relapses in CanL is crucial, ensuring timely treatment initiation, thereby preventing disease progression and improving outcomes [17]. Prompt intervention may not only reduce the parasite burden [51], lowering the risk of transmission, but also mitigate complications, enhancing the quality of life for infected dogs. Therefore, regular monitoring and assessment of risk factors prove essential for early detection of relapses and effective intervention in CanL clinical cases.

The findings of this study contribute to our understanding of the risk factors associated with clinical relapses requiring leishmanicidal treatment in CanL. By elucidating the roles of clinical scores, plasma protein components, HCT, and IFAT titers through model development, our study provides valuable insights for clinicians and researchers aiming to optimize treatment strategies and improve outcomes in CanL clinical management and decision-making.

## Conclusions

This study reveals important clinicopathological variables associated with an increased risk of clinical relapses requiring leishmanicidal treatment in CanL-sick dogs, providing valuable insights for clinicians and researchers. Dogs exhibiting higher total clinical scores, low A/G ratio, hypoalbuminemia, hyperbetaglobulinemia, anemia, and high IFAT titers are at increased risk of requiring leishmanicidal treatment due to a clinical relapse, underscoring the importance of monitoring these parameters in dogs with CanL. The contrasting findings between the two models highlight the complexity of factors influencing treatment decisions in this disease, emphasizing the need for tailored clinical management strategies to improve outcomes for sick dogs. Future studies with larger sample sizes and prospective designs are warranted to validate our findings and enhance our understanding of the factors influencing relapses in CanL.

### Supplementary Information

Additional file 1: Table S1. Clinical scoring system used in this study. Adapted from Miró et al. 2009.

Additional file 2. RScript for statistical analysis used in this study.

## Data Availability

The raw data and R script with the specific codes used in this study will be available upon request.

## Abbreviations

CanL: Canine leishmaniosis
IFAT: Indirect immunofluorescence antibody test
PCR: Polymerase chain reaction
PE: Protein electrophoresis
A/G ratio: Albumin/globulin ratio
GLMM: Generalized linear mixed-model
OR: Odds ratio
CI: Confidence interval
AIC: Akaike information criterion
VIF: Variance inflation factor
HCT: Hematocrit
WBC: White blood cell count
PLT: Platelet count
CS: Clinical score
R2: Coefficient of determination
